# Are we forgetting to carry out serum protein electrophoresis as part of diagnosis workup?

**DOI:** 10.1186/s12876-022-02477-6

**Published:** 2022-09-04

**Authors:** Mariana Barros Marcondes, Cíntia Mitsue Pereira Susuki, Newton Key Hokama, Paula de Oliveira Montandon Hokama, Felipe Aguera Oliver, Paulo Sergio Chaib, Xingshun Qi, Fernando Gomes Romeiro

**Affiliations:** 1 Internal Medicine Department, Botucatu Medical School, São Paulo State University (UNESP), Av. Prof. Montenegro, S/N - Distrito de Rubião Junior, Botucatu, São Paulo 18618-687 Brazil; 2 Radiology Department, Botucatu Medical School, São Paulo State University (UNESP), Botucatu, Brazil; 3 Surgery and Orthopedics Department, Botucatu Medical School, São Paulo State University (UNESP), Botucatu, Brazil; 4 Department of Gastroenterology, General Hospital of Northern Theater Command (Formerly General Hospital of Shenyang Military Area), Shenyang, People’s Republic of China

**Keywords:** Serum protein electrophoresis, Diarrhea, Immunodeficiency, Agammaglobulinemia, Common variable immunodeficiency, Case report

## Abstract

**Background:**

Common variable immunodeficiency (CVID) is a rare disease that affects children and adults and is often difficult to diagnose. Despite being one of the most frequent causes of immunodeficiency, involving gastrointestinal (GI), respiratory, and hematological systems, the disease onset can have heterogeneous and intermittent symptoms, frequently leading to diagnostic delay. GI symptoms are common and can include diarrhea, but the asymptomatic periods lead to overlooking the recurrent pattern. The same can occur with respiratory infections, thus delaying CVID suspicion. The starting point for CVID diagnosis is the decreased gamma globulin levels in serum protein electrophoresis (SPE), also observed through direct immunoglobulin’s dosage.

**Case presentation:**

The patient is a 38 years-old man who had intermittent diarrhea and recurrent airway infections for 19 years, but the CVID diagnosis was achieved only after SPE was carried out. At that time, he was already malnourished, and developed other complications related to CVID in a short period.

**Conclusions:**

SPE is readily available and inexpensive, but is not part of the laboratory approach in diarrhea. According to the case presented herein, it can be useful for patients with recurrent infections or other clues of the disease.

## Background

Rare diseases are part of a doctor's practice [1], requiring strategies to make the diagnosis in time and relying on simple approaches whenever possible. The diagnosis of a rare disease typically requires strikingly abnormal data, such as pathognomonic findings, or prior knowledge of a typical data set [2]. Even focusing on the most prevalent diseases, the application of organizational charts or clinical diagnosis decision support systems is helpful to avoid overlooking severe diseases [3]. The need for complex tests such as specific imaging, as well as complex biochemical or molecular dosages, can be relevant barriers to the diagnosis. Thus, simple tests that point to a disease or that can rule out another one are important tools to avoid diagnostic delay, influencing the prognosis and the patients’ quality of life [4]. This case report exemplifies that the diagnosis of a rare disease would have been easier if a routine laboratory test, the serum protein electrophoresis (SPE), had been performed earlier.

Common variable immunodeficiency (CVID) comprises a heterogeneous group of clinical and immunological phenotypes associated with varied symptoms and diverse impact on the compromised patients, who may be children or adults [5]. It is the most common symptomatic primary immunodeficiency [6]. CVID frequently presents a diagnostic challenge and should be considered in the differential diagnosis of patients with recurrent bacterial airway infections, autoimmune disease, and/or gastrointestinal manifestations [7]. The prevalence varies worldwide, but the highest rates were reported in Finland, reaching 6 to 7/100,000 [8].

The discovery of very low gamma globulin levels in SPE is the starting point for CVID diagnosis [5]. When associated with clinical findings, it can readily point to the diagnosis. Gastrointestinal (GI) involvement is reported in 9 to 20% of CVID patients [9]. The CVID diagnostic criteria are the presence of decreased levels of at least two isotypes (IgG and IgA or IgM). IgG is typically below 500 mg/dL and IgA is markedly reduced or not detectable in most cases. IgM is also below the normal range in up to 80% of patients. Flow cytometric analysis of lymphocyte subpopulations is the final diagnostic step, including total T, B and natural killer cells. It is important to diagnose late manifesting X-linked agammaglobulinemia (B cells < 0.1%) or combined immunodeficiencies (CD4 cells < 200/μl) [10].

Intermittent or chronic diarrhea, with malabsorption and abdominal pain, is the most common CVID clinical GI manifestation [7], and is caused by infections or inflammation [10]. Frequent causes include *Giardia lamblia*, *Salmonella*, *Campylobacter*, *Yersinia* and *C. difficile* [11], and the inflammation seems to be more common in the colon than in the upper GI tract. The manifestations and histologic findings may resemble autoimmune conditions such as Crohn’s disease, ulcerative colitis or celiac disease [7].

Auto-antibodies against gastric parietal cells are also detectable in CVID patients with atrophic gastritis. *Helicobacter pylori* (Hp) infection has been associated with atrophic gastritis in these patients, increasing the incidence of CVID-associated gastric cancer, typically diagnosed in patients younger than the overall gastric cancer population. Such patients can develop moderately to poorly differentiated intestinal-type adenocarcinoma, with a high number of intratumoral lymphocytes [12]. Recent data suggest declining risks due to the widespread use of antibiotic treatment, possibly due to Hp eradication [7].

It is noteworthy that GI manifestations increase the mortality risk of CVID patients by 2.7 to 4-fold [13]. Therefore, it is important to recognize the disease in time, especially in patients with diarrhea or malabsorption syndromes associated with hypogammaglobulinemia when other causes are excluded [14].

## Case description

A 38-year-old man was hospitalized due to intermittent diarrhea, weight loss and recurrent respiratory infections. The diarrhea was intermittent, with recurrent episodes in the prior nineteen years. He was unemployed and made a living from collecting recyclable material from household waste to sell in junkyards. Since the symptoms had begun, he sought medical help just a few times, when they became severe. The diarrheic periods caused 5 to 6 daily bowel movements, with no blood, fat or worms in the stool. The most severe episodes lasted up to 10 days and were eventually accompanied by vomiting, fever and weight loss, resembling infections or parasitic infestations. Despite a weight loss of 35 kg, there were no triggering factors such as medications or specific foods, and he had no family members with similar symptoms.

The medical investigation of his intermittent diarrhea had commenced about 15 years previously, when the first diagnostic hypothesis was Crohn's disease. However, he had missed follow-up, preventing further investigation. After seeking medical help in other hospitals for many years, he was finally attended again in our hospital 2 years ago due to the same symptoms, when he was already malnourished and weighed 40 kg (body mass index = 16.2 kg/m^2^). At that time, he also complained of a daily cough that had begun concomitantly with the diarrhea. The cough led to sputum production, and eventually to the elimination of some blood streaks. The episodes lasted from 1 to 3 weeks and were usually treated with antibiotics. Consequently, he had received many different antibiotics since the onset of these symptoms. He never had had dyspnea and was sure that his respiratory condition had not worsened. Besides malnutrition, no other relevant changes were noticed on physical examination. Tuberculosis tests had been performed through sputum examinations and chest X-ray exams, and had ruled out this possibility.

Infectious and parasitic diseases were ruled out through stool and blood tests. Esophagogastroduodenoscopy (EGD) showed atrophic gastritis and duodenitis. The gastric biopsies revealed chronic gastritis, mucosal atrophy and intestinal metaplasia due to Hp infection. Duodenal biopsies showed chronic duodenitis, mucosal atrophy and intraepithelial lymphocytosis. Colonoscopy displayed only a few shallow, confluent ulcers up to 5 mm in the terminal ileum, with no typical signs of Crohn’s disease in the histological evaluation. The colon and rectum were normal and celiac disease antibody tests were negative.

**Fig. 1:**
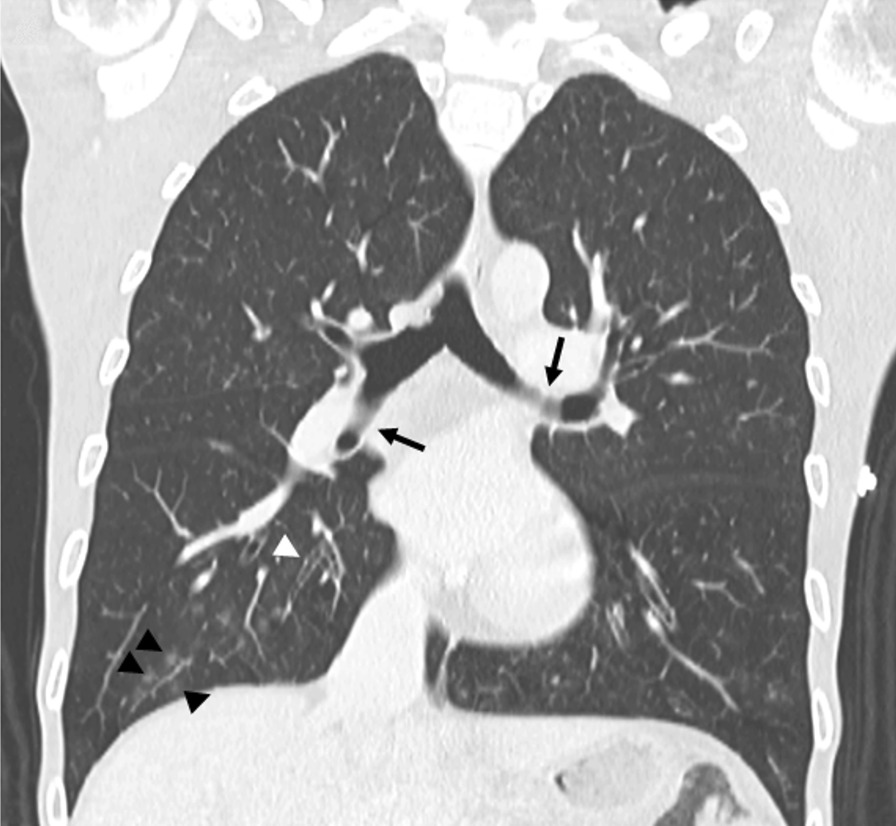
Chest computed tomography showing mucus hypersecretion (black arrows), thickening of the peri-bronchiolar walls (white arrowheads) and a “tree-in-bud” pattern reflecting bronchiolar mucoid impaction with additional involvement of adjacent alveoli (black arrowheads)

The respiratory investigation also began ruling out infectious diseases. No tuberculosis bacilli, fungi or cancer cells were found in his sputum samples. Chest computed tomography (Fig. 1) showed mucus hypersecretion (black arrows), thickening of the peri-bronchiolar walls (white arrowheads) and a ‘tree-in-bud’ pattern due to bronchiolar mucoid impaction involving the adjacent alveoli (black arrowheads). Transbronchial biopsy of the right upper lobe, where a ground-glass alveolar infiltrate was noted, revealed neutrophilic exudate and lymphocyte infiltrate, with no sign of tuberculosis, fungi or cancer.

**Fig. 2:**
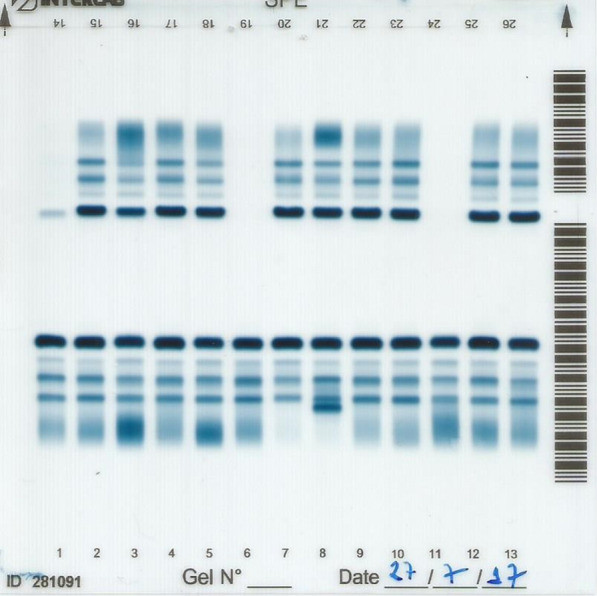
Serum protein electrophoresis. The numbers represent the electrophoretic run of different patients. When the sample is applied and the electric current is turned on, the serum proteins migrate differentially. Then, they are fixed and stained in blue. The most stained band is albumin, followed by alpha 1 globulin, alpha 2 globulin, beta globulin and gamma globulin. Column 6 shows a normal pattern of gamma globulin intensity and width. Column 7 is from the case reported, showing a marked reduction in the gamma globulin region. Column 8 was obtained from a patient with a monoclonal peak just after the beta globulin band and reduced gamma region. Column 3 displays a polyclonal gamma globulin augmentation (polyclonal hypergammaglobulinemia). After staining, the results are read by spectrometry to quantify each band

Serum protein electrophoresis (SPE) (Fig. 2) showed a marked gamma globulin reduction (arrow). The gamma globulin dosage was 0.15 g/dL (normal values: 0.6 to 2 g/dL). The beta globulin dosage was 0.55 g/dL (normal values: 0.69 to 1.57 g/dL), albumin 3.69 g/dL (normal values: 3 to 5 g/dL), alpha 1 globulin 0.21 g/dL (normal values: 0.1 to 0.38 g/dL) and alpha 2 globulin 0.60 g/dL (normal values: 0.46 to 1.14 g/dL).

Immunoglobulin A, G and M dosages were significantly reduced. IgA was 40 mg/dl (normal values: 70 to 400 mg/dL) while IgG was 270 mg/dL (normal values: 700 to 1600 mg/dL) and IgM was 25 mg/dl (normal values: 40 to 230 mg/dL). Peripheral blood lymphocyte count performed through flow cytometry showed B lymphocyte decrease to 2.12% (normal values: 5 to 15%). The CD4 count was 539 cells/µl (normal values: 410 to 1590 cells/µl), whereas that of CD8 was 1101 cells/µl (normal values: 190 to 1140 cells/µl). Therefore, the CD4/CD8 ratio was 0.47 (normal values: 0.9 to 2.6). The percentage of NK lymphocytes was 7.36% (normal values: 5 to 15%).

After confirming the CVID diagnosis, the patient missed follow-up again and returned two years later, complaining of epigastric pain. EGD and biopsies revealed poorly differentiated intestinal type gastric adenocarcinoma in the posterior wall of the gastric body. He was operated on, but did not tolerate the chemotherapy and passed away one year after the surgery, due to pneumonia and septic shock.

## Discussion and conclusions

SPE is an inexpensive and widely available test that measures specific serum proteins according to their migratory profile in a standardized electric field [15]. The differences among the migration pattern of albumin and the four globulin fractions (alpha 1, alpha 2, beta and gamma) can be clearly observed. Quantification of each protein is based on the spectrophotometric data and the amount of total protein. The test is mainly required to search for monoclonal gammopathies, which narrow the gamma globulin peak. Another typical finding in the gamma fraction is a polyclonal increase, especially in chronic inflammatory diseases, such as infections, systemic lupus erythematosus, or liver/intestinal diseases. Hypogammaglobulinemia, an unusual finding, was pivotal for the diagnosis of CVID in the presented case. Of note, it was done only after many other invasive and expensive tests, when infections were ruled out and immunosuppression was suspected.

CVID encompasses a group of disorders of primary antibody production failure [5, 13, 16]. Hypogammaglobulinemia is the hallmark of the disease, mostly diagnosed in adults between 20 and 40 years old in association with other puzzling manifestations, including recurrent bacterial infections and chronic gastrointestinal complaints resembling autoimmune or neoplastic diseases [17]. Other causes of hypogammaglobulinemia should be excluded, such as neoplasms (chronic lymphocytic leukemia and lymphomas), nephrotic syndrome, medications (immunosuppressants, anticonvulsants, chemotherapy, rituximab) and other primary genetic immunodeficiencies. After documenting the hypogammaglobulinemia through SPE, quantitative determination of serum immunoglobulins is the next step to diagnose CVID. It is noteworthy that the patient described herein had no hypoalbuminemia, even when he was clearly malnourished. As mentioned above, the CVID diagnostic criteria are the presence of decreased levels of at least two isotypes (IgG and IgA or IgM). In our case, the three immunoglobulins were decreased. The patient had normal T lymphocyte counts, but the diminished CD4/CD8 ratio suggested a relative decrease in helper T lymphocytes. His results were similar to other cases with a relative loss of T-cell function, including a lack of circulating CD4 + cells [16]. He also had a decreased B lymphocyte count, but this is not a diagnostic criterion. Although CVID patients have an intrinsic B cell defect, there is a significant heterogeneity in involvement of the B-cell subsets [5].

The patient had recurrent infections affecting both upper airways and lungs, with CT findings certainly caused by the disease. The CT images are one of the strengths of this report, as well as the SPE comparisons between the patient and other interesting cases. The main limitation is that the patient missed follow-up for 2 years, preventing any specific treatment. He regretted when the cancer was discovered and he was already too ill. Regarding respiratory findings, CVID patients can have repeated infections, obstructive pulmonary disease and bronchiectasis. Pulmonary interstitial infiltrates on high-resolution chest CT appear as reticulonodular changes and fibrosis, and resemble ground glass [12]. The initial workup in our case was focused on ruling out parasitic infestation, tuberculosis, fungal diseases, lung cancer, acquired immunodeficiency, celiac disease, pernicious anemia and inflammatory bowel diseases. Since most of the lymphoid tissue of the human body belongs to the GI tract, GI disorders are clinically challenging in CVID, not only for their prevalence in this population (9–20% of patients), but also because they are difficult to treat [18].

Typically, in CVID-associated small bowel villous atrophy, there are no antibodies against tissue transglutaminase, endomysium or gliadin, thus contributing to the negative results of these tests [18]. A gluten-free diet can reduce the symptoms in approximately 20% of CVID patients, but wheat avoidance can contribute to further weight loss. As observed in the case presented, the risk of gastric cancer is high, especially when the patient is chronically infected by Hp, which favors cancer development. Since the infection is an additional cause, the bacteria eradication could reduce the cancer risk, but the patient missed follow-up every time he was attended, thereby delaying the CVID diagnosis and preventing Hp eradication. Moreover, the broad range of nonspecific manifestations can lead to an average delay of 6–7 years in CVID diagnosis [17]. Of note, although SPE is inexpensive and highly available [19], it is not part of the chronic diarrhea workup [20]. Therefore, we suggest that SPE should be part of the diagnosis workup for patients with recurrent infections, especially when congenital immunodeficiency is suspected, to avoid diagnostic delay in CVID cases.

## Data Availability

The datasets analyzed during the current study are not publicly available because they belong to the patient medical chart, but are available from the corresponding author on reasonable request.

## Abbreviations

CVID: Common variable immunodeficiency
EGD: Esophagogastroduodenoscopy
GI: Gastrointestinal
Hp: *Helicobacter pylori*
SPE: Serum protein electrophoresis
